# Pre-Chamber Ignition Mechanism: Simulations of Transient Autoignition in a Mixing Layer Between Reactants and Partially-Burnt Products

**DOI:** 10.1007/s10494-018-9960-0

**Published:** 2018-07-17

**Authors:** Jennifer A. M. Sidey, Epaminondas Mastorakos

**Affiliations:** Hopkinson Laboratory, Engineering Department, University of Cambridge, Cambridge, CB2 1PZ UK

**Keywords:** Pre-chamber ignition, Flamelet, Natural gas engines

## Abstract

The structure of autoignition in a mixing layer between fully-burnt or partially-burnt combustion products from a methane-air flame at *ϕ* = 0.85 and a methane-air mixture of a leaner equivalence ratio has been studied with transient diffusion flamelet calculations. This configuration is relevant to scavenged pre-chamber natural-gas engines, where the turbulent jet ejected from the pre-chamber may be quenched or may be composed of fully-burnt products. The degree of reaction in the jet fluid is described by a progress variable *c* (*c* = taking values 0.5, 0.8, and 1.0) and the mixing by a mixture fraction *ξ* (*ξ* = 1 in the jet fluid and 0 in the CH_4_-air mixture to be ignited). At high scalar dissipation rates, *N*_0_, ignition does not occur and a chemically-frozen steady-state condition emerges at long times. At scalar dissipation rates below a critical value, ignition occurs at a time that increases with *N*_0_. The flame reaches the *ξ* = 0 boundary at a finite time that decreases with *N*_0_. The results help identify overall timescales of the jet-ignition problem and suggest a methodology by which estimates of ignition times in real engines may be made.

## Introduction

The ignition of a reactive mixture when brought in contact with hot (or burnt) gases is one of the canonical problems in combustion science and has been studied extensively, with the first perhaps being by Marble and Adamson [1] who estimated analytically the ignition time in the one-dimensional and initially infinitely-thin mixing layer between reactants and their adiabatically burnt products. When the hot products region has finite size, the solution to this problem in general also gives the conditions under which a flame may begin to propagate in the reactive mixture given the size of the region of the hot gases and their temperature, and the quantification of such conditions has been the usual focal point. This results in an expression for the minimum ignition energy (MIE), often estimated as the amount of energy needed to raise to the adiabatic flame temperature a region of characteristic size of the order of the laminar flame thickness (with a numerical factor depending on geometry and the Lewis number; some pertinent results are reviewed in Refs. [2, 3]).

These analyses typically assume no flow and hence do not take velocity gradients into account. However, the behaviour of reaction zones under the action of stretch caused by aerodynamic strain can be different from their behaviour when unstretched. Hence, for instance, a non-premixed counterflow layer between hot air and cold fuel may not autoignite if the strain rate is too high [4], the MIE in an already premixed homogeneous mixture will increase if there is turbulence in the flow [3, 5], and a cylindrical ”spark” (i.e. a hot gas column), as when a hot inert gas jet is injected into premixed reactants, may not lead to ignition if its diameter or velocity is too high [6–8].

An important practical application of the latter problem is the pre-chamber ignited engine [9–11]. In case the orifice in the pre-chamber is small, only hot gases escape the pre-chamber and not a vigorous reaction zone, but still ignition may occur. The pre-chamber may be at the same or a higher equivalence ratio than the main chamber. Due to the quenching at the orifice, the possibility that the gases escaping are partially burnt cannot be discounted. An experimental study investigating these phenomenon is given in Ref. [8]. A related observation is that an explosion may be transmitted through a gap between two plates, as may be present in flanges in pressure vessels, even if the gap is significantly below the nominal quenching distance of the mixture and hence only a fast jet of hot gases escape the vessel [12].

A canonical problem simplifying these practical configurations is a strained layer between a reactive mixture and hot combustion products, of the same or some other mixture, including the possibility that these products are not fully reacted. This paper discusses numerical simulations of this problem focusing on the ignition time as a function of strain rate and degree of reaction completion in the hot products stream, highlighting a procedure that may be useful for estimating ignition times in pre-chamber engines when the orifice is smaller than the minimum needed to allow a flame to be ejected. This diffusion-reaction system is also relevant to MILD combustion [13–16] and the extinction of counterflow premixed flames against hot products [17–19].

## Method

To investigate the autoignition of lean mixtures by burned or partially burned hot combustion products, a laminar, transient, counterflow flame was simulated with an in-house Conditional Moment Closure code [20], with spatial dependence removed, hence denoted as “0DCMC” in the rest of this paper. Species mass fractions, *Y*_*α*_, were calculated in mixture fraction (*ξ*) space with Eq. 1:

1 $$ \frac{\partial Y_{\alpha}}{\partial t}=N(\xi)\frac{\partial^{2} Y_{\alpha}}{\partial \xi^{2}} +\omega_{\alpha} $$

The temperature was calculated from the total enthalpy that was conserved (straight line in *ξ*-space). The scalar dissipation rate, *N*(*ξ*), is not constant across *ξ*, but comes from the well-known inverse error function profile parametrised by the maximum value *N*_0_, so that *N*(*ξ*) = *N*_0_*G*(*ξ*), with *G*(*ξ*) = *exp*(− 2[*erf*^− 1^(2*ξ* − 1)]^2^). The results from the solution to these equations are identical to laminar transient flamelet calculations with *Le*_*α*_ = 1 and the given distribution of scalar dissipation. The GRI Mech 3.0 chemical mechanism was used [21].

**Fig. 1:**
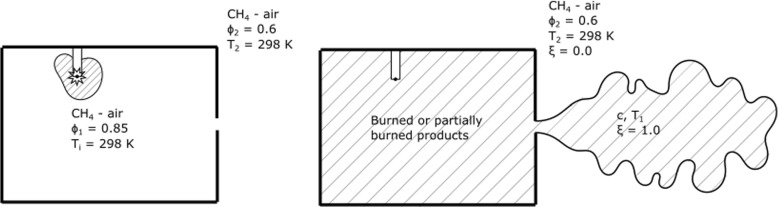
A schematic of the jet ignitor system in the spark stage (left) and jet ignitor stage (right). This work is concerned with the ignition of the mixture outside the ignitor chamber by the burned or partially burned products in the hashed region in the ignitor stage

**Fig. 2:**
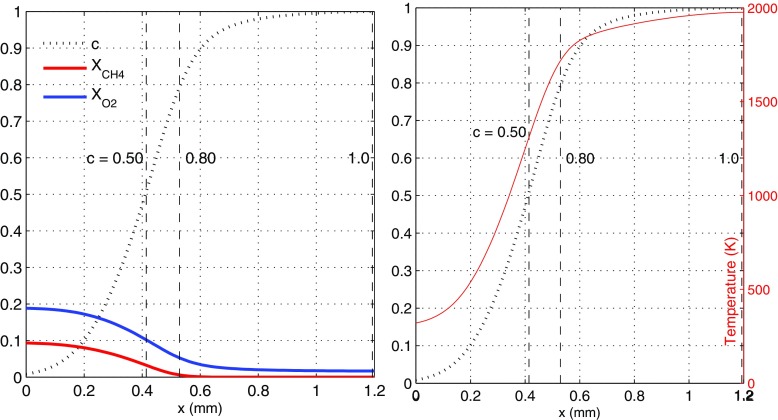
Species (left), temperature (right), and progress variable across the *ϕ* = 0.85 laminar methane-air premixed flame at 1 bar, *T* = 300 K. Values of *c* = 0.50, 0.80, and 1.00 are marked with a dashed line

A premixed methane-air flame at atmospheric pressure and *T*_*i*_ = 300 K (*ϕ* = 0.85) was used to generate burned or partially burned products in COSILAB [22], with the same chemical mechanism, representing the jet fluid emerging from the ignitor as fully or partially burned gas. A progress variable, *c*, was used to define a composition and temperature of burned or partially burned products based on the *O*_2_ content of the premixed flame solution. A *c* = 0.50, for example, corresponds to a composition and temperature in which half of the premixed *O*_2_ has been consumed by the premixed flame (Fig. 1). A graphical representation of the variable *c* is given in Fig. 2.

Mixtures investigated range from *c* = 0.50, representing only a partially burned jet, to *c* = 1.0, which are fully burned adiabatic hot combustion products. The composition and temperatures of each of these burned product conditions is summarised in Table 1. While only what are considered key chemical species are shown in Table 1, note that all 53 species are included in the burned or partially burned products. These mixtures were then set to the right boundary condition in the 0DCMC code (*ξ* = 1.0). The left boundary (*ξ* = 0) was a methane-air mixture at *T*_*i*_ = 300 K and equivalence ratio 0.6, to use values representative of jet-ignited gas engines. This system offers a simplified representation of the chemical evolution in the configuration schematically shown in Fig. 1.

**Table 1:** Jet composition and temperature

*c*	*T*_1_	*X*_*CH*4_	*X*_*O*2_	*X*_*OH*_	*X*_*CH*2 *O*_
	[K]				
0.5	1310	3.24 × 10^− 2^	0.102	2.48 × 10^− 4^	9.55 × 10^− 4^
0.8	1717	6.02 × 10^− 3^	0.053	2.32 × 10^− 3^	4.97 × 10^− 4^
1.0	1975	3.49 × 10^− 12^	0.017	7.77 × 10^− 3^	1.13 × 10^− 10^

## Results and Discussion

**Fig. 3:**
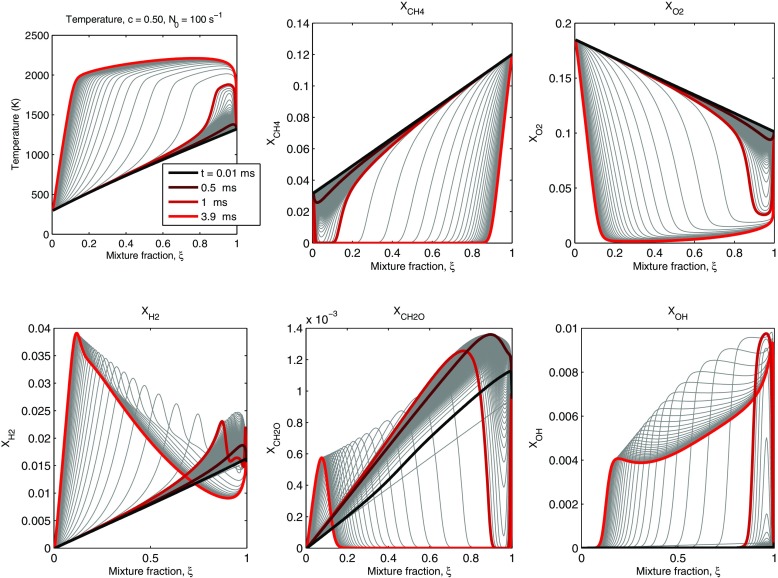
Temperature and species profiles (CH_4_, O_2_, H_2_, CH_2_O, and OH) during autoignition of *c* = 0.5 cases and *N*_0_ = 100 *s*^− 1^

Figure 3 shows the distributions in *ξ* of the temperature and various mass fractions, for different times. The case chosen is for *c* = 0.5 and for a relatively low peak scalar dissipation *N*_0_, as representative of a situation that will lead to autoignition. The temperature is highest at *ξ* = 1 initially, but autoignition of the layer occurs very close to this mixture fraction (around *ξ* = 0.95) and eventually a reaction front begins to propagate across *ξ*-space. Ignition of the premixed flame at the *ξ* = 0 mixture will happen once this front reaches this boundary. This instant depends on the autoignition time, but also on the speed of the front movement. The former will be discussed later in detail. The speed of the front movement across *ξ* increases with *N*_0_ (not shown here), consistent with results in other non-premixed autoignition systems [4]. From the curves in Fig. 3, and defining as “ignition” the instant when the maximum temperature across the layer reaches 2000 K, we could say that ignition occurs just after 1 ms. The corresponding distributions of the fuel and oxygen show the consumption of these species. Formaldehyde is initially produced, and suddenly consumed at ignition, while the OH radical grows rapidly at ignition and fills the whole *ξ*-space, surviving at large values even after ignition in the post-flame region of the propagating front. The *H*_2_ intermediate, which exists in finite quantities at *ξ* = 1 due to the fact that the species there correspond to partially-burnt products, gets further produced by the oxidation of CH_4_ but also consumed post-flame. Note that these simulations were run with constant values of the species mass fractions at *ξ* = 0 and *ξ* = 1. This does not allow chemical evolution at these values of mixture fraction, which constrains the development of the flame, especially as it moves towards *ξ* = 0. However, this is far removed from the region of ignition and hence the autoignition times calculated here should be reasonable.

**Fig. 4:**
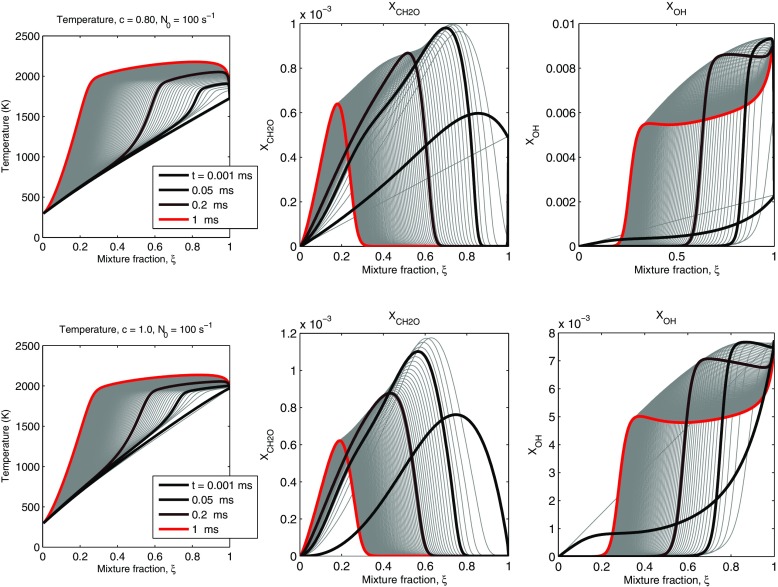
Temperature and selected species (CH_2_O and OH) profiles during autoignition of *c* = 0.8 (upper row) and *c* = 1 (lower row) cases for *N*_0_ = 100 *s*^− 1^

Similar evolutions for the *c* = 0.8 and *c* = 1 cases are shown in Fig. 4. The autoignition now is quicker, but broadly speaking the evolution of the species is similar. Autoignition is associated with the sudden consumption of the accumulated CH_2_O, and the front reaches the *ξ* = 0 boundary in less than 1 ms.

**Fig. 5:**
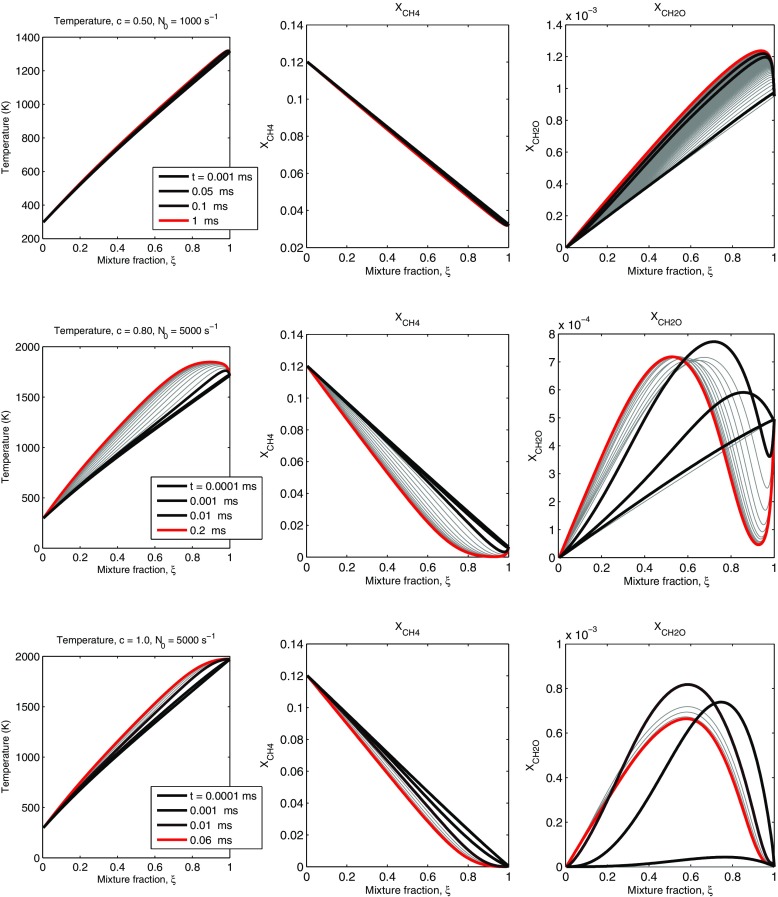
Temperature and selected species (CH_4_ and CH_2_O) profiles during autoignition of *c* = 0.5 and $N_{0} = 1000 \textit {s}^{-1}$ (upper); *c* = 0.8, $N_{0}= 5000 \textit {s}^{-1}$ (middle); and *c* = 1, $N_{0}= 5000 \textit {s}^{-1}$ (lower)

Figure 5 shows temperature, CH_4_, and CH_2_O evolutions for the three cases for high scalar dissipation. A qualitatively different behaviour is evident compared to the low *N*_0_ cases. First, for *c* = 0.5, autoignition does not occur, but the long-time behaviour of the system is a state of slow reaction, very little temperature rise, minor CH_4_ consumption, and a substantial presence of CH_2_O everywhere. The high mixing rate does not allow the system to autoignite. The higher *c* cases also reach a steady-state where there is no propagating front across *ξ*-space, and hence no chance of igniting the *ξ* = 0 mixture. Due to the higher temperatures reached, there is some CH_4_ removal, but still the temperature rise is small and a steady-state with substantial CH_2_O everywhere is reached.

**Fig. 6:**
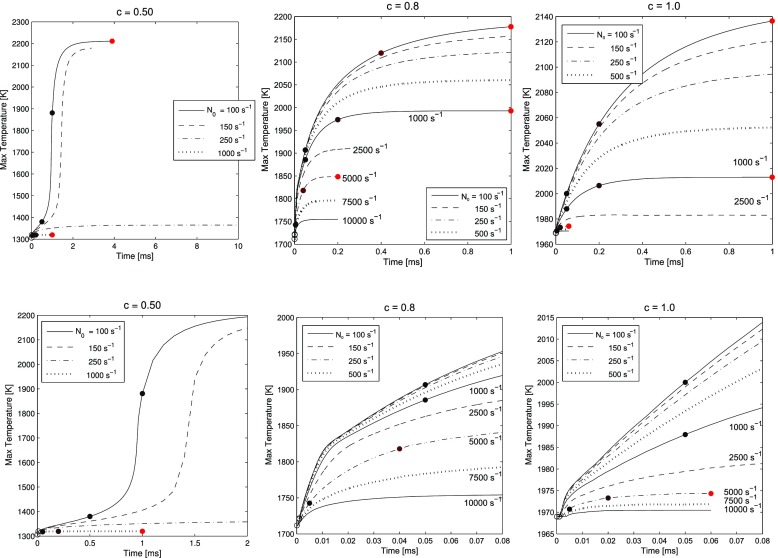
Maximum temperature across *ξ*-space as a function of time, for all cases and for various scalar dissipations *N*_0_. The top row shows a larger scale on the x-axis (time, *t* [ms]) while the bottom row shows the early stages of ignition for each case

Figure 6 shows the maximum temperature across the solution domain for all the cases studied. It is evident that for *c* = 0.5, a conventional autoignition event is presented, with an inflection point in the temperature *vs.* time curve and a large jump in temperature from the initial value. It is also evident that a qualitatively different behaviour arises when *N*_0_ is high: the maximum temperature reaches a steady state at a very small temperature increment from the initial value, which can be associated with a slow oxidation process. In contrast, when the products are at a higher *c*, there is a gradual evolution to the maximum value, which becomes lower as *N*_0_ increases. Since high *N*_0_ means large rates of mixing in *xi*-space, a high value suggests chemistry is not fast enough to keep with mixing, and hence the temperature rise across *ξ*-space is smaller. Note the absence of a sudden transition at high *c* values.

Similar smooth ignition behaviour with the absence of a clearly defined inflection point was observed by Sidey et al. [14] in plug flow reactor simulations with hot combustion products. In this work, the presence of hot combustion product and intermediate species in the high temperature oxidiser caused an elongated ignition event which merged with an equilibrium reaction at the cold boundary. The presence of this elongated ignition event may be a marker for a transition into a preheated and dilution combustion regime. Sidey and Mastorakos also investigated steady counterflow flames with hot combustion products as an oxidiser and found that, as levels of dilution reached high levels, counterflow systems did not show evidence of sudden extinction, even at high rates of strain, but instead behaved with a monotonic S-Shaped curve [23]. This behaviour may also be observed here, with a chemically-frozen state reached at high *N*_0_ for the *c* = 0.8 an *c* = 1.0, but no sudden transition to such a state noticeable in comparison with the *c* = 0.5 case, which occurs between *N*_0_ = 150 *s*^− 1^ and 250 *s*^− 1^.

**Fig. 7:**
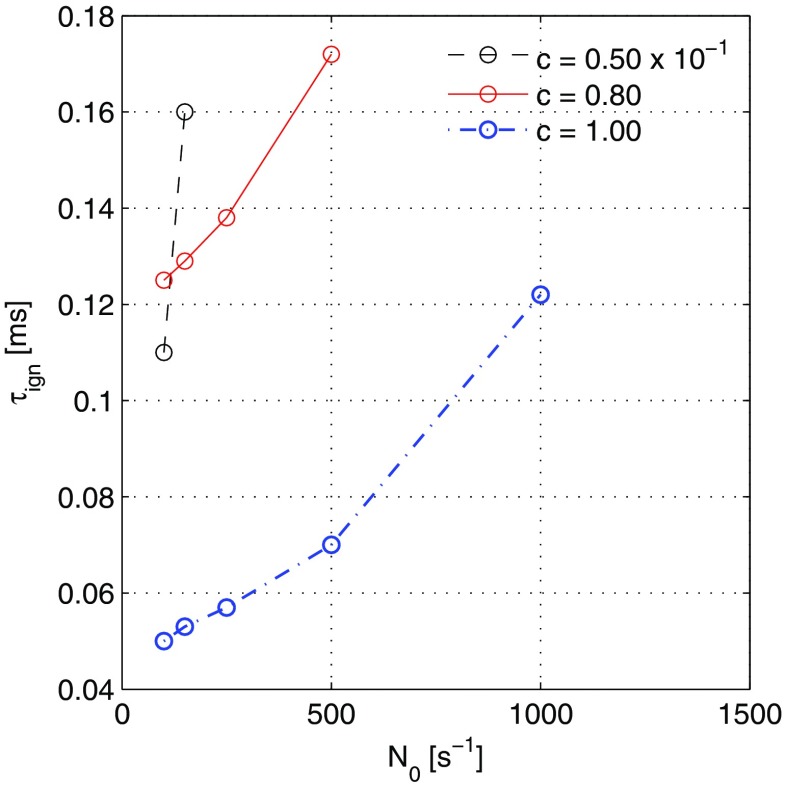
Ignition delay time (*τ*_*ign*_, ms) as a function of scalar dissipation rate for *c* = 0.50, 0.80, and 1.00 cases. Note that *c* = 0.50 results are plotted x 10^− 1^

Figure 7 compiles the autoignition times for the various cases as a function of scalar dissipation. It is evident that: (i) the *c* = 0.5 cases autoigntite much later than the others; (ii) the case with adiabatic equilibrium products (*c* = 1) autoignites the earliest, although *still* at a finite time (which will be different for engine conditions); and (iii) the autoignition time increases with scalar dissipation. In an jet-ignited engine, the use of orifices with small diameter leads to higher *N* and may also lead to *c* < 1 in the emerging products, both factors detrimental to autoignition. However, high *N* values may not last too long in the jet and hence proper evaluation of the autoignition time in an engine environment requires recourse to the full turbulent reacting flow problem and modelling by models such as CMC or the PDF method.

## Conclusions

The structure of autoignition of a methane-air mixture of a lean equivalence ratio and fully-burnt or partially-burnt combustion products from a methane-air flame at *ϕ* = 0.85 has been investigated with transient diffusion flamelet calculations. These results are investigated in a context with relevance to scavenged pre-chamber natural-gas engines, where a turbulent jet emerges from a pre-chamber as either quenched partially-burnt products or fully-burnt products. A progress variable, *c*, is used to describe the degree of reaction of the jet. Jet values of *c* = 0.5, *c* = 0.8, and *c* = 1.0 are investigated here. At high scalar dissipation rates, *N*_0_, ignition does not occur and a chemically-frozen steady-state condition emerges at long times. However, ignition is observable at very high scalar dissipation rates for cases with a higher temperature jet (*c* = 0.8 or *c* = 1.0). Ignition delay time increases with *N*_0_ and decreases with *c*. These results suggest that very small jet orifices, which will increase *N* and lead to quenched products (*c* < 1.0) may inhibit ignition. The results help identify overall timescales of the jet-ignition problem and suggest a methodology by which estimates of ignition times in real engines may be made.
